# Supplementary material for: The effect of producer groups on the productivity and technical efficiency of smallholder cocoa farmers in Ghana
Source: PLoS One. 2023 Dec 11;18(12):e0294716. doi: 10.1371/journal.pone.0294716 (PMC10712861; doi:10.1371/journal.pone.0294716)
Supplement: Attachment — Submitted filename: Comments on Manuscript PONE-D-23-18150.docx [file pone.0294716.s001.docx]

**My comments on the Manuscript Number PONE-D-23-18150**

The paper presents a good study for using the truncated normal distribution stochastic frontier model and an endogenous treatment regression model to analyse the impact of coco producer groups' on the farmers' technical efficiency in Ghana . It is perceived that, farm size, labour, and capital significantly and positively affect the partial elasticity of production. Age, education, use of hybrid cocoa, involvement in off-farm jobs, extension access, and producer group membership significantly affect inefficiency. Moreover, the results of the endogenous treatment regression model revealed that, the producer group membership significantly impacts technical efficiency and yield. The study recommends that producer groups should be made accessible to farmers, and Policymakers should play big role in the formation and strengthening of producer groups. Overall, this interesting well written study tackles an important issue of how to improve the technical efficiency of coco farmers through encouraging producer group (both cooperatives and farmer associations). I think there is the need for this study to allow research and policy to reach far-reaching conclusions. However, I have several comments that can be considered to enhance the publication potential:

Introduction section: it is preferable to add subheading to highlight the coco production in Ghana, because some results is not understandable. For instance, the average age of cocoa trees is higher for non-group farmers than for group producers, likewise, the percentage numbers of farmers who used hybrid crops did. Otherwise, give some information on the recommended age for replacement, cooperative group and associations in addition to others important practices related to the study.

**Data and Methodology**:

Study area: The author (s) did not provide any background quantitative information on the Ahafo-Ano South and South-west districts in the Ashanti region of Ghana. Please write a paragraph on the region and explain why this was the only region included in this study. It is preferable to include a map.

Research methodology: Yield (Y) is generally defined as a function of inputs (X) applied in producing the crop (i.e., Y=f(X)). It is not a function of cost. Need revision or justification.

**Recommendations**: it is clear from the results that, non-group farmers used much labour whereas, group farmers goes for more capital inputs (more agrochemical and equipment. It is also clear that, group members has better yield and technical efficiency than non-group. Moreover, credit has no statistical impact. **The question here from where do group members get money to finance their additional capital costs**, the answer might be from off-farm income or their society. Thus, off farm job negatively affect efficiency and yield while farm size has a negative influence on cocoa yield. I think you need to stress on this point in the recommendations.

**References:** need revision and update to 2023; some references were not mentioned in the references list (e.g. Mohammed et al, 2011, Asibey-Bonsu, 2012). Other quoted wrongly like Addai et al., 2014 written in the text et. al., instead of (Addai and Owusu).
